# Phase‐Contrast Magnetic Resonance Imaging Identifies Low Cerebrospinal Fluid Velocity at the Foramen Magnum in Small Breed Dogs With an Enlarged Ventricular System

**DOI:** 10.1111/jvim.70197

**Published:** 2025-09-26

**Authors:** Sarah Hubler, Christina Precht, Gertraud Schüpbach‐Regula, Veronika M. Stein, Daniela Schweizer

**Affiliations:** ^1^ Division of Clinical Radiology, Department of Clinical Veterinary Medicine Vetsuisse Faculty, University of Bern Bern Switzerland; ^2^ Veterinary Public Health Institute, Vetsuisse Faculty, University of Bern Liebefeld Switzerland; ^3^ Division of Clinical Neurology, Department of Clinical Veterinary Medicine Vetsuisse Faculty, University of Bern Bern Switzerland

**Keywords:** canine, CSF flow disturbance, CSF obstruction, hydrocephalus, phase‐contrast, ventriculomegaly

## Abstract

**Background:**

In small breed dogs, enlarged ventricles of the brain are a common finding on magnetic resonance imaging (MRI). In humans, enlarged lateral ventricles are usually the consequence of mesencephalic aqueduct stenosis. Cerebrospinal fluid (CSF) velocity measurements indicating obstruction are lacking in dogs.

**Objectives:**

Measure CSF velocity in small breed dogs with ventricular enlargement.

**Animals:**

Velocity of CSF in 17 small breed dogs with enlarged ventricles and 8 small breed dogs with normal‐sized ventricles was measured by phase‐contrast MRI at the mesencephalic aqueduct, foramen magnum (FM) and second cervical vertebra (C2).

**Methods:**

Peak systolic (PSV) and diastolic (PDV) velocity, peak velocity (PV), difference between peak systolic and diastolic velocity (DPV), average velocity (AV) and maximum average velocity (MAV) were measured.

**Results:**

Dogs with enlarged ventricles had lower PDV, PV, AV, and MAV at the dorsal subarachnoid space of the FM compared with dogs without enlargement (*p* < 0.05). At the ventral subarachnoid space of FM, moderate decreases in PDV, PV, DPV, AV, and MAV were found with increasing severity of ventricular enlargement.

**Conclusion:**

Ventricular enlargement may be associated with or result in altered CSF flow dynamics, particularly decreased velocity at the craniocervical junction. This relationship may, in turn, reflect underlying structural changes, such as skull shape or craniocervical abnormalities. Therefore, enlarged ventricles in small breed dogs should be considered pathological findings.

**Abbreviations**
AV: average velocity
C2: second cervical vertebral body
CI: cephalic index
CSF: cerebrospinal fluid
DPV: difference between systolic and diastolic peak velocity
FM: foramen magnum
FOV: field of view
MAV: maximum average velocity
PC‐MRI: phase‐contrast magnetic resonance imaging
PDV: peak diastolic velocity
PSV: peak systolic velocity
PV: peak velocity
ROI: region of interest
SAS: subarachnoid space
TE: echo time
TR: repetition time
VENC: velocity encoding
Vh/Bh: ventricle to brain height ratio
VV/BV: ventricle to brain volume ratio

## 1 Introduction

The popularity of small breed dogs has increased in recent years [1]. In these dogs, the prevalence of distention of the ventricular system is notable [2, 3, 4, 5]. When this condition occurs without accompanying clinical signs, it is termed ventriculomegaly [2, 4]. Conversely, when clinical signs are present, hydrocephalus is diagnosed [3, 5, 6, 7]. Currently, there is no proven explanation for the frequent occurrence of enlargement of the ventricular system in small breed dogs. Stenosis of the mesencephalic aqueduct with fusion of the rostral colliculi is the most common cause of congenital hydrocephalus [3]. Decreased cranial capacity and brachycephaly leading to decreased absorption of cerebrospinal fluid (CSF) through lymphatic pathways have been proposed as potential causes of ventricular enlargement [2, 4]. Additionally, malformations at the craniocervical junction are thought to alter craniospinal CSF flow dynamics because of obstruction of the foramen magnum, further contributing to this condition [2].

The maintenance of a constant volume of blood, brain tissue, and CSF within the skull results in a pulsatile bidirectional CSF flow [8, 9, 10, 11, 12]. During cardiac systole, CSF flows compensatorily caudally, whereas it flows in a rostral direction during diastole [10]. Its velocity can be measured using phase‐contrast magnetic resonance imaging (PC‐MRI) [10, 13]. This procedure is based on a bipolar gradient with opposite polarity applied after the initial radiofrequency pulse [10, 13, 14]. Doing so generates a net phase shift of moving spins that is directly proportional to its flow velocity [10, 13, 14]. Encoding velocity (VENC) is an important parameter of a PC‐MRI examination because it adjusts the magnitude of the bipolar gradient [10, 14]. Aliasing artifact results from underestimating VENC, whereas overestimating it leads to a low signal [10].

In humans, PC‐MRI is used to evaluate conditions such as hydrocephalus and Chiari malformation [10, 13, 14, 15, 16]. Pediatric patients with obstructive hydrocephalus have decreased CSF peak velocity at the site of obstruction [17]. In veterinary medicine, PC‐MRI has been used to measure CSF velocity in healthy dogs [18, 19, 20] and shows that CSF peak velocity is higher in dogs < 2 years of age, male dogs, and those weighing > 20 kg compared with dogs weighing < 10 kg [20]. Dogs with ventriculomegaly and internal hydrocephalus have increased CSF velocities at the level of the mesencephalic aqueduct compared with normal controls [21]. It is unknown if dogs with enlarged ventricles show additional abnormalities in CSF velocity measured by PC‐MRI, but in most Cavalier King Charles Spaniels with syringomyelia, PC‐MRI images identified obstruction of CSF flow at the level of the foramen magnum (FM) [22].

We aimed to investigate if small breed dogs with enlargement of at least one internal CSF space show signs of CSF velocity alteration and if the location of CSF flow disturbance caused by obstruction can be identified using PC‐MRI. We assumed that a lack of phase shift at any time point during systole and diastole indicates obstruction. We hypothesized that the CSF velocity of small breed dogs with enlargement of at least one internal CSF space is significantly decreased at the site of obstruction.

## 2 Materials and Methods

Our study was conducted as a prospective case–control study including small breed dogs < 10 kg in body weight that were divided into two groups based on the magnetic resonance imaging (MRI) appearance of the ventricular system. The Cantonal Veterinary Office of Bern approved the use of client‐owned dogs for the study (BE47/2022).

Client‐owned small breed dogs undergoing MRI of the brain for diagnostic purposes were included in the study. Additionally, with the owners' consent, two small breed dogs that had undergone general anesthesia for suspected disc herniation at the thoracolumbar spine because of acute onset of paraplegia had an MRI examination of the brain exclusively for our study. Exclusion criteria were parenchymal lesions causing a mass effect with displacement of adjacent brain structures and brain atrophy as defined based on the thickness of the interthalamic adhesion [23, 24]. Dogs with alterations at the craniocervical junction, such as Chiari‐like malformation or keyhole‐shaped foramen magnum as well as syringomyelia were not excluded.

### 2.1 Anesthesia

All dogs were fasted for at least 6 h before anesthesia. The anesthesia protocol was adjusted to the individual dog. Premedication (IM or IV) included medetomidine (0.002–0.01 mg/kg, Domitor ad us. vet., Provet AG, Switzerland) or acepromazine (0.005–0.01 mg/kg, Prequillan ad us. vet., Arovet AG, Switzerland) combined with an opioid such as butorphanol (0.1–0.4 mg/kg, Morphasol‐10 ad us. vet., Dr. E Graeub AG, Switzerland) or methadone (0.2 mg/kg, Methadon Streuli 10 mg/mL, Streuli Pharma AG, Switzerland). An IV catheter (jelco, 18G to 22G, Smiths Medical ASD, inc., USA) was aseptically placed in a cephalic or saphenous vein.

All dogs were preoxygenated for 3 min with an oxygen flow of 2 L/min using a facemask. Anesthesia was induced with propofol (Propofol 1%, MCT Fresenius, Fresenius Kabi AG, Switzerland) titrated to effect to allow orotracheal intubation. Depending on the individual case, a co‐induction with ketamine (1–2 mg/kg, Ketasol 100, ad us. vet., Dr. E Graeub AG, Switzerland), midazolam (0.1–0.2 mg/kg, Midazolam Sintetica 5 mg/mL, Sintetica S.A., Switzerland) or lidocaine (1 mg/kg, Lidocain 2% Streuli, Streuli Pharma AG, Switzerland) was performed. Anesthesia was performed using a rebreathing system and maintained with sevoflurane (Sustane Sevoflurane ad us. vet., Piramal Critical Care, USA) administered to effect and vaporized in an oxygen‐air admixture (inspired fraction of oxygen of 50%). Dogs were mechanically ventilated by volume‐control ventilation (tidal volume of 10 mL/kg), with respiratory rate adjusted to maintain end‐tidal CO_2_ between 30 and 40 mmHg. The dogs received IV fluid administration of Ringer‐acetate (2–5 mL/kg/h, Ringer Acetat Fresenius i.v., Fresenius Kabi AG, Switzerland). Dogs were monitored clinically for eye position, presence or absence of reflexes and muscle tone, and instrumentally using pulse oximetry (saturation and pulse rate), capnography, inhalant gas analysis, spirometry, core temperature, and non‐invasive blood pressure. Anesthetic complications were managed on a case‐by‐case basis. After the PC‐MRI measurements, anesthesia was either prolonged for further clinical interventions, or the dogs were recovered.

### 2.2 Magnetic Resonance Imaging

The MRI studies were performed using a 3 Tesla MRI scanner (Magnetom Vida, Siemens, Erlangen, Germany). Dogs were placed in sternal recumbency with their heads in a flexed position. Flexion of the head was documented by an angle between a line from the tuberculum sellae to the basion and a line between the craniodorsal and caudodorsal borders of the vertebral body of the axis (angle of head position) [25]. Care was taken to avoid pressure on the jugular veins. Dogs with a small head were positioned in an 18‐channel flex coil (Ultraflex Small 18, Siemens). Dogs with a larger head were positioned in an 18‐channel knee coil (Tx/Rx Knee 18, Siemens). Pulse‐gated phase‐contrast sequences were performed using a magnetic resonance compatible a peripheral pulse unit placed on the tongue (PPu_2G Peripheral Pulse Unit, Siemens, Erlangen, Germany).

**FIGURE 1:**
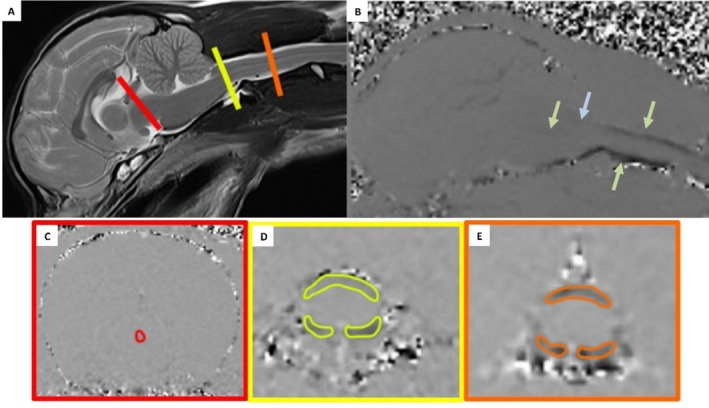
T2 weighted midsagittal image (A) of a 3 years old male Chihuahua diagnosed with idiopathic epilepsy. The red, yellow and orange lines mark the locations where the transverse phase‐contrast measurements were carried out (red = mesencephalic aqueduct, yellow = foramen magnum, orange = second cervical vertebra). (B) Midsagittal phase‐contrast sequence of the brain and upper cervical spine. The dark signal dorsal and ventral to the spinal cord and cerebellum represents the cerebrospinal fluid (CSF) phase shift due to moving protons (green arrows). The blue arrow points to a focal loss of signal ventral to the obex extending caudally to the foramen magnum. (C) transverse phase‐contrast image at the level of the mesencephalic aqueduct. The red circle indicates mesencephalic aqueduct. (D) transverse phase‐contrast image at the level of the foramen magnum. The yellow circles indicate the dorsal and ventral subarachnoid space of the foramen magnum. (E) transverse phase‐contrast image at the level of the second cervical vertebra. The orange markings indicate the dorsal and ventral subarachnoid space of the second cervical vertebra.

A dorsal 3D‐T1 sequence was performed for evaluation of the ventricular system. Four PC‐MRI sequences were performed: one mid‐sagittal plane through the brain and cranial cervical spine and transverse planes at the [1] mid‐ampulla of the mesencephalic aqueduct, [2] 1 mm caudal to the occipital condyles (FM) and [3] at the mid body of the second cervical vertebral body (C2; Figure 1) [18]. The PC‐MRI measurement time varied with heart rate, ranging from 12 to 16 min. Sequence parameters are listed in Table 1.

**TABLE 1:** Listed parameters for the sequences used for the evaluation of the ventricular system and cerebrospinal fluid velocity.

	Dorsal 3D‐T1 sequence		Sagittal PC‐MRI sequence		Transverse PC‐MRI sequences	
	Flex coil	Knee coil	Flex coil	Knee coil	Flex coil	Knee coil
TR (ms)	2400	2500	28.5	32.4	34.8	35.2
TE (ms)	3.52	3.31	10.2	12.03	12.91	12.91
Flip‐angle (°)	8	8	10	10	10	10
FOV (mm)	100 × 100	125 × 125	100 × 100	160 × 160	120 × 120	100 × 100
Voxel size (mm)	0.63 × 0.63 × 0.79	0.65 × 0.65 × 1				
Average	1	1	2	1	1	1
Heart phases			40	40	40	40
Matrix			115 × 144	205 × 256	205 × 356	205 × 256
Slice thickness (mm)			6	5	6	6
VENC (cm/s)			3	3	2	2

Abbreviations: FOV, field of view; TE, echo time; TR, repetition time; VENC, velocity encoding.

Phase contrast images were qualitatively analyzed for a brightness shift at the suspected location of the CSF space. Image planning was reevaluated if no bright to dark shift was present. If aliasing occurred, the VENC was increased up to 5 cm/s, and if the signal was low, VENC was decreased to 2 cm/s.

### 2.3 Image Analysis

The three‐dimensional (3D) T1‐weighted sequence of the brain of all dogs was evaluated for lateral ventricular height, brain height, and the angle of head position. A T1 3D MPRAGE sequence was evaluated for cephalic index, which is the maximum cranial width divided by the maximum cranial length × 100, [25] using DeepUnity Diagnost (DeepUnity Diagnost 1.1.0.1, DH Healthcare GmbH, Germany). To calculate the ventricle‐to‐brain height ratio (Vh/Bh), the height of the brain and mean height of right and left lateral ventricles were measured at the level of the interthalamic adhesion on transverse images [26]. The ventricle‐to‐brain volume ratio (VV/BV) was calculated based on manual segmentation of all internal CSF spaces and brain parenchyma on a T1 3D MPRAGE sequence using ITK‐SNAP (ITK‐SNAP version 3.6.0, www.itksnap.org) [27].

**FIGURE 2:**
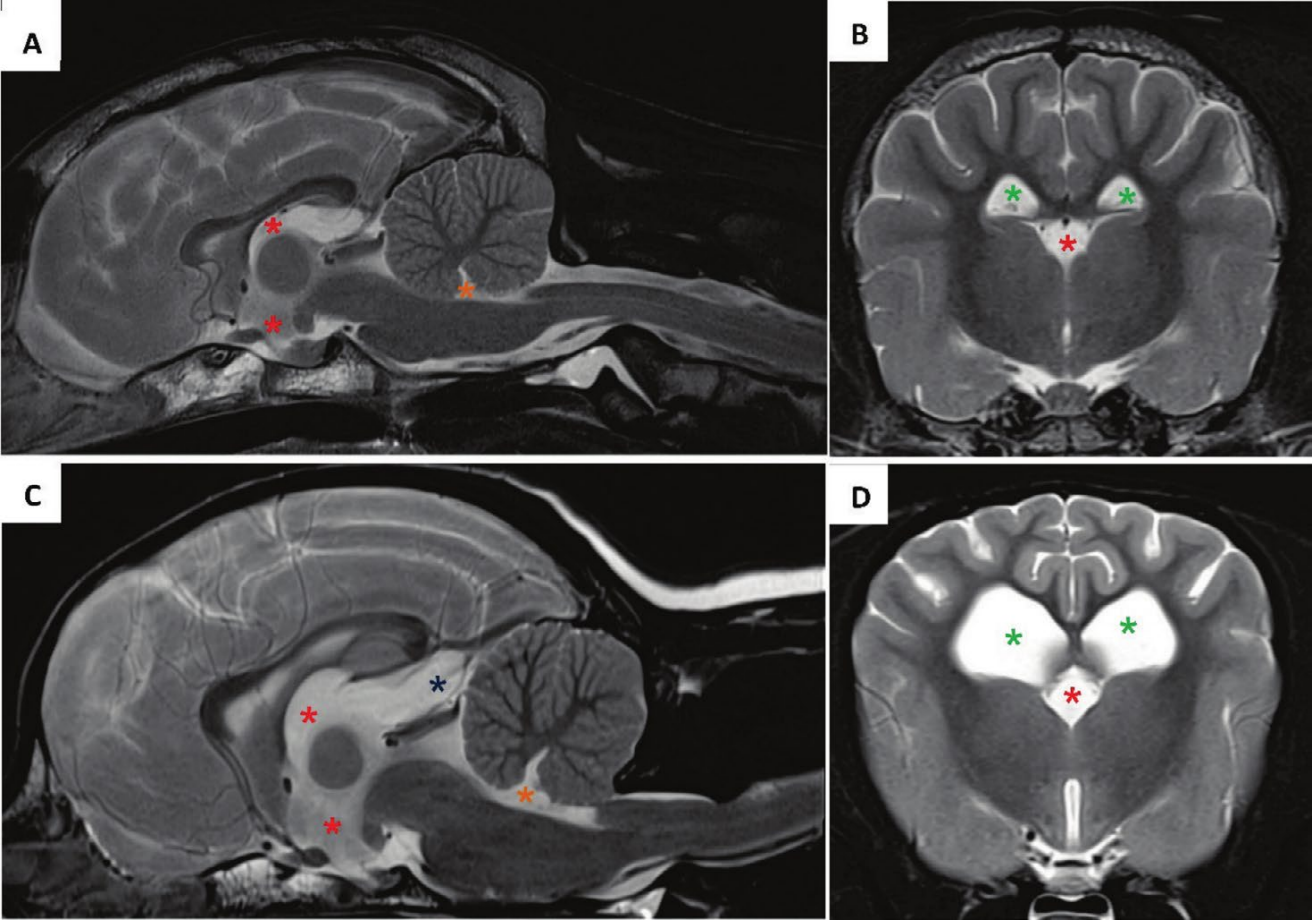
T2‐weighted midsagittal (A, C) and transverse images (B, D) at the level of the interthalamic adhesion of a dog assessed as without enlargement of the internal CSF spaces (A, B) and of a dog assessed as enlarged internal CSF spaces (C, D). The green stars indicate location of lateral ventricles, red stars the third ventricle, the orange stars the fourth ventricle and the blue star a supracollicular fluid accumulation.

Categorization of the dogs was based solely on the size of their ventricular system, independent of their clinical presentation or neurological status. Dogs were categorized by a board‐certified radiologist into Group 1 exhibiting dilatation of at least one internal CSF space and Group 2 with no enlargement of the ventricular system (Figure 2). The lateral ventricles were classified as normal‐sized (Vh/Bh = 0%–14%) and enlarged (Vh/Bh > 14%) [26]. The classification of the third and fourth ventricles into normal‐sized and enlarged, as well as the presence of a supracollicular fluid accumulation, was subjectively made.

Using an external workstation (syngo MR XA31, MAGNETOM Vida, Siemens Healthcare GmbH, Erlangen, Germany), phase‐contrast (PC) images were quantitatively analyzed for

1. peak diastolic velocity: highest positive velocity measured in a single pixel of the region of interest (ROI) during the entire cardiac cycle
2. peak systolic velocity: highest negative velocity measured in a single pixel of the ROI during the entire cardiac cycle
3. peak velocity [17]: highest velocity measured in a single pixel of the ROI during the entire cardiac cycle
4. difference between diastolic and systolic velocity
5. average velocity: mean velocity of all pixels in the ROI during the entire cardiac cycle
6. maximal average velocity: maximum mean velocity of all pixels in the ROI during the entire cardiac cycle

The first author, instructed and supervised by a board‐certified radiologist, manually drew a ROI within the CSF spaces on the transverse phase images within the mesencephalic aqueduct and the dorsal subarachnoid space and ventral left and right subarachnoid space of the FM and C2 (Figure 1) [18, 20]. The mean value of the right and left ventral subarachnoid space of the FM and C2 was defined as the ventral subarachnoid space of the FM and C2 [20].

The first and last author descriptively evaluated the sagittal phase images for lack of phase shift in the CSF spaces during diastole and systole (Figure 1). A focal loss of signal on phase contrast images at any time point during the cardiac cycle was identified as decreased CSF velocity indicating obstruction [22].

### 2.4 Statistical Analysis

Statistical analyses were performed using NCSS (NCSS 2022 Statistical Software (2022). NCSS LLC. Kaysville, Utah, USA, ncss.com/software/ncss). A Mann–Whitney *U* test was used to assess differences in numerical variables (age, body weight, CSF velocity data, Vh/Bh, VV/BV, CI). A chi‐squared test was used to test a potential association of sex between groups. A Spearman rank correlation test was used to investigate correlation.

## 3 Results

Group 1 consisted of 17 small breed dogs with an enlargement of at least one internal CSF space (Table 2). Breeds represented were six Chihuahuas, four Pomeranians, two Yorkshire Terriers, two Cavalier King Charles Spaniels, and one each of a French Bulldog, Maltese, and Bolonka Zwetna. Reasons for undergoing MRI of the brain for diagnostic purposes were seizures (eight dogs), cervical pain (two dogs), ataxia (one dog), vestibular syndrome (one dog), episodes of suspected pain (one dog), chronic otitis (one dog), behavioral changes (one dog), apathy and persistent miosis (one dog) and increased scratching (one dog). Dogs were diagnosed with idiopathic epilepsy or movement disorder (seven dogs), syringomyelia (five dogs), metabolic‐toxic encephalopathy (two dogs), C4–5 intervertebral disc extrusion (one dog), otitis media and interna (one dog) and facial and vestibulocochlear neuropathy (one dog).

**TABLE 2:** Affected internal CSF spaces in the 17 dogs with enlargement of at least one internal CSF space.

Number of dogs	Enlarged ventricles			
	Lateral ventricles	Thid ventricle	Fourth ventricle	Supracollicular fluid accumulation
5 (29.4%)	x	x	x	x
4 (23.5%)	x	x	x	
1 (5.9%)	x	x		x
1 (5.9%)	x	x		
1 (5.9%)	x		x	x
1 (5.9%)	x		x	
1 (5.9%)		x	x	x
1 (5.9%)		x		x
1 (5.9%)			x	x
1 (5.9%)			x	

Group 2 consisted of eight small breed dogs without enlargement of the ventricular system. Three of the dogs were mixed breed dogs. The other dogs were a Bichon Frisé, Biewer Terrier, Pomeranian, Jack Russell Terrier, and Dachshund. Reasons for undergoing MRI of the brain for diagnostic purposes were seizures (three dogs), clinical signs suggestive of ear discomfort (one dog), tetraparesis (one dog), and vestibular ataxia (one dog). Two dogs with disc herniation had an additional MRI of the brain specifically for our study. Dogs were diagnosed with intervertebral disc extrusion at the thoracolumbar spine (two dogs), idiopathic epilepsy or movement disorder (two dogs), spinal lesion at the upper thoracic spine (one dog), otitis media and interna (one dog), otitis externa (one dog) and metabolic‐toxic encephalopathy (one dog).

Table 3 summarizes descriptive data and statistical results for both groups. No differences were found in mean age, sex distribution, or CSF velocity measurement success (*p* = 0.62, *p* = 0.68, all *p* > 0.05, respectively). Similarly, no difference was identified in the success rate of CSF velocity measurements comparing the two groups (all *p* > 0.05). However, dogs with enlargement of at least one internal CSF space had lower mean body weight (4.0 kg vs. 6.2 kg, *p* = 0.01), larger dilatation of the lateral ventricles (Vh/Bh;19.0% vs. 9.4%, *p* = 0.00), larger dilatation of the ventricular system (VV/BV; 6.5% vs., 1.4%, *p* = 0.00), and a higher cephalic index indicating more severe brachycephaly (83.11 vs. 73.3, *p* = 0.00).

**TABLE 3:** Descriptive data and number of dogs with successful CSF velocity measurements of the dogs with enlargement of at least one internal CSF space (Group 1) and dogs without enlargement of the ventricular system (Group 2). A higher ventricular height to brain height ratio (Vh/Bh) indicates enlarged lateral ventricles, a higher ventricular volume to brain volume ratio (VV/BV) indicates enlarged internal CSF spaces. The degree of brachycephaly in a dog is directly reflected by its cephalic index, with more brachycephalic dogs exhibiting higher cephalic index. The red stars * indicate significant different parameters between the two groups.

			Group 1 (*n* = 17)	Group 2 (*n* = 8)	*p*
Sex distribution			7 females 10 males	4 females 4 males	0.68
Mean age ± SD (range) (years)			4.7 ± 3.1 (0.5–11.6)	5.0 ± 2.9 (0.4–9)	0.62
Mean body weight ± SD (range) (kg)			4.0 ± 1.7 (1.7–8.8)*	6.2 ± 2.1 (3.8–9)*	0.01*
Vh/Bh ± SD (range) (%)			19.0 ± 6.7 (9.27–37.25)*	9.39 ± 2.24 (6.12–12.23)*	0.00*
VV/BV ± SD (range) (%)			6.5 ± 4.3 (1.27–15.47)*	1.42 ± 0.57 (1.10–2.62)*	0.00*
Cephalic index ± SD (range)			83.11 ± 5.9 (73.66–93.61)*	73.32 ± 5.0 (64.1–80.06)*	0.00*
Successful PC measurements	All locations		*n* = 7 (41.2%)	*n* = 3 (37.5%)	0.86
	Sagittal		*n* = 9 (53.0%)	*n* = 4 (50%)	0.9
	All transverse		*n* = 7 (41.2%)	*n* = 3 (37.5%)	0.86
	Mesencephalic aqueduct		*n* = 13 (76.5%)	*n* = 6 (75%)	0.94
	FM	Ventral	*n* = 13 (76.5%)	*n* = 7 (87.5%)	0.52
		Dorsal	*n* = 8 (47.1%)	*n* = 5 (62.5%)	0.47
	C2	Ventral	*n* = 14 (82.4%)	*n* = 6 (75%)	0.67
		Dorsal	*n* = 12 (70.6%)	*n* = 5 (62.5%)	0.69
	None		*n* = 3 (17.6%)	*n* = 1 (12.5%)	0.74

Abbreviations: C2, second cervical vertebra; FM, foramen magnum; *n*, number of dogs; SD, standard deviation; Vh/Bh, lateral ventricle to brain height ratio; VV/BV, ventricle to brain volume ratio.

### 3.1 Quantitative Analysis of CSF Velocity

The CSF velocity parameters quantitatively measured at the level of the mesencephalic aqueduct and ventral and dorsal subarachnoid space at the level of the FM and C2 were compared between the two groups (Table 4 and Data S1).

**TABLE 4:** Cerebrospinal fluid (CSF) velocity measurements of the dogs with enlargement of at least one internal CSF space (Group 1) and dogs without enlargement of the ventricular system (Group 2) at the mesencephalic aqueduct and ventral and dorsal subarachnoid space within the foramen magnum (FM) and second cervical vertebra (C2). The red stars * indicate significant differences in CSF velocity parameters between both groups.

		Mesencephalic aqueduct	FM		C2	
			Ventral subarachnoid space	Dorsal subarachnoid space	Ventral subarachnoid space	Dorsal subarachnoid space
Mean peak systolic velocity ± SD (SEM) (cm/s)	Group 1	−0.50 ± 0.36 (0.10)	−0.77 ± 0.39 (0.11)	−0.45 ± 0.16 (0.06)	−1.04 ± 0.59 (0.16)	−0.77 ± 0.54 (0.16)
	Group 2	−0.49 ± 0.57 (0.25)	−0.83 ± 0.41 (0.15)	−0.82 ± 0.45 (0.20)	−1.03 ± 0.54 (0.22)	−0.90 ± 0.54 (0.24)
	*p*	0.62	0.76	0.11	0.90	0.46
Mean peak diastolic velocity ± SD (SEM) (cm/s)	Group 1	0.70 ± 0.29 (0.08)	0.77 ± 0.19 (0.05)	0.59 ± 0.27* (0.10)	0.80 ± 0.31 (0.08)	0.90 ± 0.47 (0.14)
	Group 2	0.71 ± 0.63 (0.26)	1.05 ± 0.23 (0.11)	0.88 ± 0.29* (0.13)	0.94 ± 0.28 (0.12)	0.97 ± 0.09 (0.04)
	*p*	0.28	0.07	0.045*	0.31	0.11
Mean peak velocity ± SD (SEM) (cm/s)	Group 1	0.75 ± 0.35 (0.10)	0.90 ± 0.31 (0.09)	0.60 ± 0.26* (0.09)	1.09 ± 0.58 (0.16)	1.04 ± 0.51 (0.15)
	Group 2	0.71 ± 0.63 (0.26)	1.11 ± 0.31 (0.12)	1.05 ± 0.47* (0.21)	1.37 ± 0.57 (0.23)	1.16 ± 0.31 (0.14)
	*p*	0.24	0.16	0.03*	0.24	0.44
Mean difference between systolic and diastolic velocity ± SD (SEM) (cm/s)	Group 1	1.12 ± 0.62 (0.17)	1.54 ± 0.51 (0.14)	1.04 ± 0.42 (0.15)	1.84 ± 0.81 (0.22)	1.66 ± 0.89 (0.26)
	Group 2	1.25 ± 1.25 (0.56)	1.87 ± 0.65 (0.25)	1.70 ± 0.68 (0.31)	1.97 ± 0.62 (0.25)	1.86 ± 0.57 (0.25)
	*p*	0.77	0.35	0.07	0.44	0.44
Mean average velocity ± SD (SEM) (cm/s)	Group 1	0.21 ± 0.10 (0.03)	0.19 ± 0.07 (0.02)	0.17 ± 0.05* (0.02)	0.19 ± 0.06 (0.02)	0.24 ± 0.14 (0.04)
	Group 2	0.22 ± 0.13 (0.05)	0.24 ± 0.04 (0.02)	0.23 ± 0.02* (0.01)	0.21 ± 0.10 (0.04)	0.20 ± 0.04 (0.02)
	*p*	0.70	0.11	0.03*	0.40	0.72
Mean max. average velocity ± SD (SEM) (cm/s)	Group 1	0.46 ± 0.20 (0.06)	0.51 ± 0.16 (0.05)	0.29 ± 0.06* (0.02)	0.72 ± 0.39 (0.10)	0.63 ± 0.28 (0.08)
	Group 2	0.46 ± 0.36 (0.15)	0.66 ± 0.28 (0.10)	0.51 ± 0.18* (0.08)	0.67 ± 0.14 (0.06)	0.62 ± 0.33 (0.15)
	*p*	0.37	0.39	0.006*	0.66	1.00

Abbreviations: C2, second cervical vertebra; FM, foramen magnum; SD, standard deviation; SEM, standard error.

**FIGURE 3:**
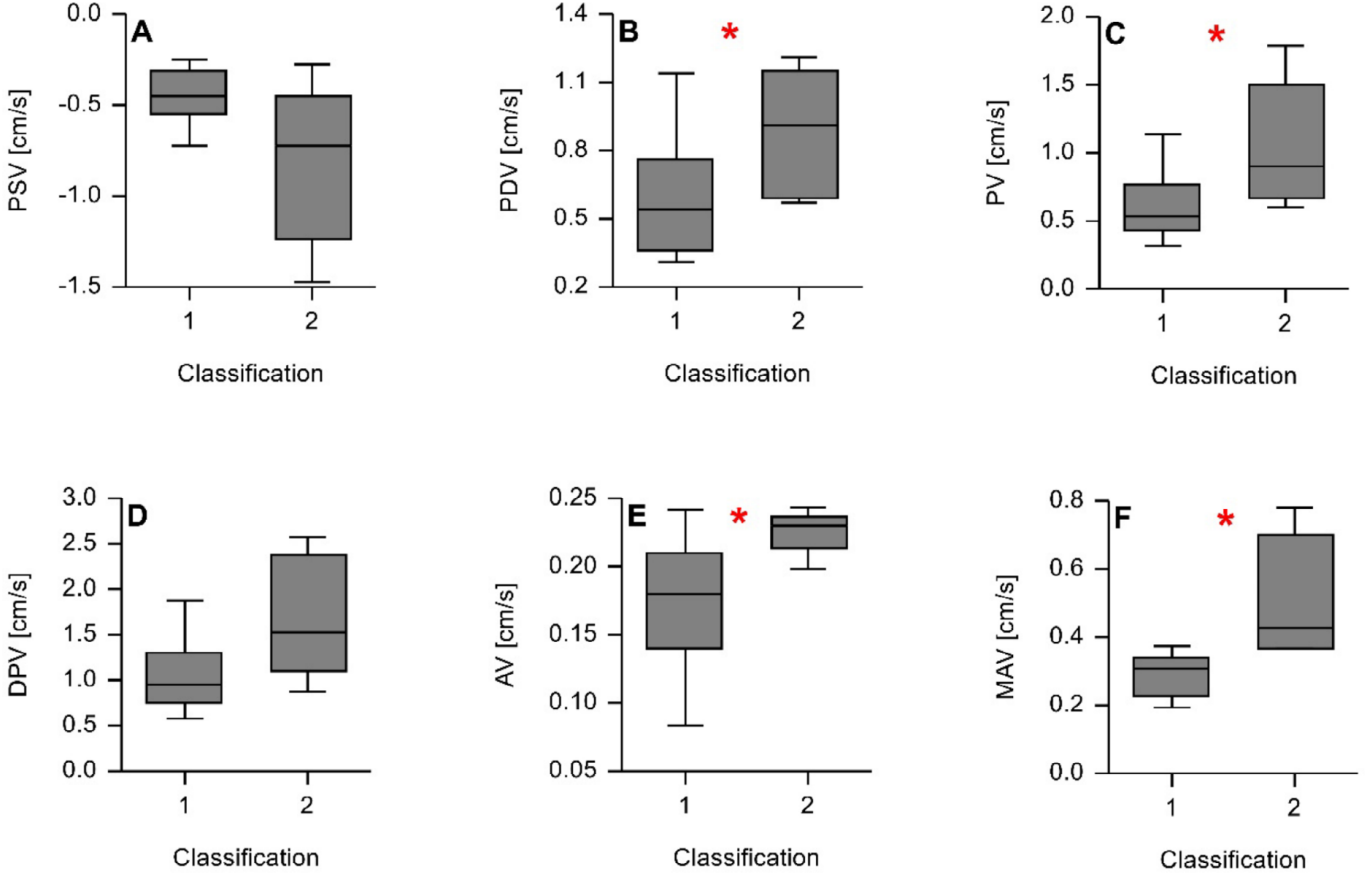
Box and whiskers plots depicting cerebrospinal fluid (CSF) velocity measurements at the dorsal subarachnoid space at the level of the foramen magnum (FM) according to classification into [1] dogs with enlargement of at least one internal CSF space and [2] dogs without enlargement of the ventricular system. The length of the whiskers is 1.5 times the interquartile range, with observations exceeding this distance considered outliers. (A) peak systolic velocity (PSV) (note that the velocity scale is negative), (B) peak diastolic velocity (PDV), (C) peak velocity (PV), (D) difference between peak systolic and diastolic velocity (DPV), (E) average velocity (AV) and (F) maximum average velocity (MAV). Due to the direction of CSF flow (negative PSV and positive PDV), DPV is lower in group 1 compared to group 2. The red stars * indicate significant differences in CSF velocity parameters between both groups.

Multiple differences in mean CSF velocity parameters were found at the level of the dorsal subarachnoid space of the FM. Dogs with an enlargement of at least one internal CSF space had lower mean peak diastolic velocity (*p* = 0.04), mean peak velocity (*p* = 0.03), mean average velocity (*p* = 0.03) and mean maximum average velocity (*p* = 0.01) compared with dogs with no enlargement of the ventricular system (Figure 3). No difference in mean peak systolic velocity (*p* = 0.11) and mean difference between diastolic and systolic velocity (*p* = 0.07) was found.

No difference was observed in any mean CSF velocity parameter within the mesencephalic aqueduct, ventral subarachnoid space at the FM, and ventral and dorsal subarachnoid space at C2 (all *p* > 0.05).

In summary, we observed decreased mean CSF velocity parameters in dogs with enlargement of at least one internal CSF space within the dorsal subarachnoid space at the level of the FM compared with dogs without enlargement of the ventricular system.

### 3.2 Correlation of CSF Velocity Parameters With Lateral Ventricle‐To‐Brain Height Ratio (Vh/Bh)

The relationship between CSF velocity parameters and enlargement of the lateral ventricles, expressed by the Vh/Bh ratio, was examined at the level of the mesencephalic aqueduct, FM, and C2 (Table 5).

**TABLE 5:** Overview of correlations between CSF velocity parameters and lateral ventricle to brain height ratio (Vh/Bh), ventricular to brain volume ratio (VV/BV) and cephalic index (CI). A correlation factor between 0.40 and 0.69 can be interpreted as a moderate correlation (**bold font**), whereas a weak correlation is described by a correlation factor between 0.10 and 0.39. No correlation is described with a correlation factor of less than 0.10.

		Lateral ventricle to brain height ratio (Vh/Bh)	Ventricular to brain volume ratio (VV/BV)	Cephalic index (CI)
Mesencephalic aqueduct (correlation coefficient (*p*))	PSV	−0.32 (0.20)	−0.25 (0.33)	0.04 (0.86)
	PDV	0.34 (0.16)	0.34 (16)	0.10 (0.69)
	PV	**0.40 (0.09)**	0.39 (0.11)	0.13 (0.60)
	DPV	0.18 (0.47)	0.23 (0.37)	−0.03 (0.92)
	AV	0.07 (0.77)	0.20 (0.42)	−0.02 (0.93)
	MAV	0.**41 (0.08)**	**0.45 (0.06)**	0.21 (0.39)
Ventral subarachnoid space within the foramen magnum (correlation coefficient (*p*))	PSV	0.21 (0.37)	0.26 (0.28)	0.31 (0.19)
	PDV	−0.38 (0.10)	**−0.42 (0.08)**	**−0.61 (0.004)**
	PV	**−0.43 (0.06)**	**−0.47 (0.04)**	**−0.60 (0.005)**
	DPV	−0.35 (0.13)	**−0.41 (0.08)**	**−0.52 (0.02)**
	AV	**−0.43 (0.06)**	**−0.48 (0.04)**	**−0.63 (0.003)**
	MAV	−0.31 (0.19)	**−0.42 (0.07)**	−0.37 (0.11)
Dorsal subarachnoid space within the foramen magnum (correlation coefficient (*p*))	PSV	0.20 (0.50)	0.12 (0.70)	**0.45 (0.13)**
	PDV	−0.34 (0.25)	−0.14 (0.66)	**−0.58 (0.04)**
	PV	−0.38 (0.19)	−0.22 (0.48)	**−0.60 (0.03)**
	DPV	−0.32 (0.28)	−0.17 (0.60)	**−0.57 (0.04)**
	AV	**−0.53 (0.06)**	−0.35 (0.27)	**−0.62 (0.02)**
	MAV	**−0.54 (0.05)**	−0.34 (0.28)	**−0.60 (0.03)**
Ventral subarachnoid space of C2 (correlation coefficient (*p*))	PSV	0.10 (0.67)	0.12 (0.61)	0.25 (0.30)
	PDV	−0.12 (0.62)	−0.20 (0.40)	**−0.40 (0.08)**
	PV	−0.25 (0.29)	−0.30 (0.20)	**−0.52 (0.02)**
	DPV	−0.14 (0.55)	−0.20 (0.41)	**−0.44 (0.05)**
	AV	−0.30 (0.20)	−0.31 (0.18)	−0.16 (0.50)
	MAV	−0.24 (0.31)	−0.32 (0.16)	**−0.45 (0.05)**
Dorsal subarachnoid space of C2 (correlation coefficient (*p*))	PSV	**0.44 (0.08)**	0.29 (0.27)	0.04 (0.89)
	PDV	**−0.52 (0.03)**	−0.33 (0.21)	−0.24 (0.36)
	PV	**−0.58 (0.02)**	**−0.45 (0.08)**	−0.09 (0.74)
	DPV	**−0.50 (0.04)**	−0.35 (0.19)	−0.07 (0.79)
	AV	0.01 (0.98)	−0.25 (0.36)	0.05 (0.85)
	MAV	−0.34 (0.18)	−0.24 (0.36)	0.04 (0.87)

Abbreviations: AV, average velocity; DPV, difference between peak systolic and diastolic velocity; MAV, maximum average velocity; PDV, diastolic velocity; PSV, Peak systolic; PV, peak velocity.

Moderately lower peak velocity (rho = −0.43, *p* = 0.06) and average velocity (rho = −0.43, *p* = 0.06) with a higher Vh/Bh ratio were found within the ventral subarachnoid space of the FM. Weak correlations between Vh/Bh ratio and peak systolic velocity (rho = 0.21, *p* = 0.37), peak diastolic velocity (rho = −0.38. *p* = 0.1), difference between diastolic and systolic velocity (rho = −0.35, *p* = 0.13) and maximum average velocity (rho = −0.31, *p* = 0.19) were found within the ventral subarachnoid space of the FM. At the level of the dorsal subarachnoid space within the FM, a moderately higher average velocity (rho = −0.53, *p* = 0.06) and maximum average velocity (rho = −0.54, *p* = 0.05) with a lower Vh/Bh ratio were found. Weak correlations between Vh/Bh ratio and peak systolic velocity (rho = 0.20, *p* = 0.50), peak diastolic velocity (rho = −0.34, *p* = 0.25), peak velocity (rho = −0.38, *p* = 0.19) and difference between diastolic and systolic velocity (rho = −0.32, *p* = 0.28) were found within the dorsal subarachnoid space of the FM.

At the dorsal subarachnoid space of C2, a higher Vh/Bh ratio was associated with moderately higher peak systolic velocity (rho = 0.44, *p* = 0.08) and moderately lower peak diastolic velocity (rho = −0.52, *p* = 0.03), peak velocity (rho = −0.58, *p* = 0.02), and difference between diastolic and systolic velocity (rho = −0.50, *p* = 0.04). Weak or no correlation between the Vh/Bh ratio and average velocity (rho = 0.007, *p* = 0.98) and maximum average velocity (rho = −0.34, *p* = 0.18) was found at the dorsal subarachnoid space of C2.

Weak or no correlations were found between most quantitatively analyzed CSF velocity parameters and the Vh/Bh ratio within the mesencephalic aqueduct and ventral subarachnoid space of C2 (all rho < 0.4 and *p* > 0.05). However, moderately higher peak velocity (rho = 0.40, *p* = 0.09) and maximum average velocity with a higher Vh/Bh ratio (rho = 0.41, *p* = 0.08) were observed at the level of the mesencephalic aqueduct.

### 3.3 Correlation of CSF Velocity Parameters With Ventricle‐To‐Brain Volume Ratio (VV/BV)

Correlation between CSF velocity parameters and enlargement of the ventricular system, expressed by the VV/BV ratio, was investigated at the level of the mesencephalic aqueduct, FM, and C2 (Table 5).

Within the ventral subarachnoid space of the FM, evidence of moderately lower peak diastolic velocity (rho = −0.42, *p* = 0.08), peak velocity (rho = −0.47, *p* = 0.04), difference between the diastolic and systolic velocity (rho = −0.41, *p* = 0.08), average velocity (rho = −0.48, *p* = 0.04) and maximum average velocity (rho = −0.42, *p* = 0.07) with a higher VV/BV ratio were found. These CSF velocity parameters decrease as the ventricular system enlarges. A weak correlation was observed between peak systolic velocity (rho = 0.26, *p* = 0.23) and VV/BV ratio within the ventral subarachnoid space of the FM.

Within the mesencephalic aqueduct, dorsal subarachnoid space of the FM and ventral and dorsal subarachnoid space of C2, weak or no correlation was measured between any CSF velocity parameter and VV/BV (all rho < 0.4 and *p* > 0.05). Exceptions were moderately higher maximum average velocity with higher VV/BV ratio (rho = 0.45, *p* = 0.06) at the level of the mesencephalic aqueduct and moderately higher peak velocity (rho = −0.45, *p* = 0.08) with lower VV/BV ratio at the level of the dorsal subarachnoid space within C2.

### 3.4 Correlation of Ventricular Size (Vh/Bh and VV/BV) and CSF Velocity Parameters With the Degree of Brachycephaly (Cephalic Index)

**FIGURE 4:**
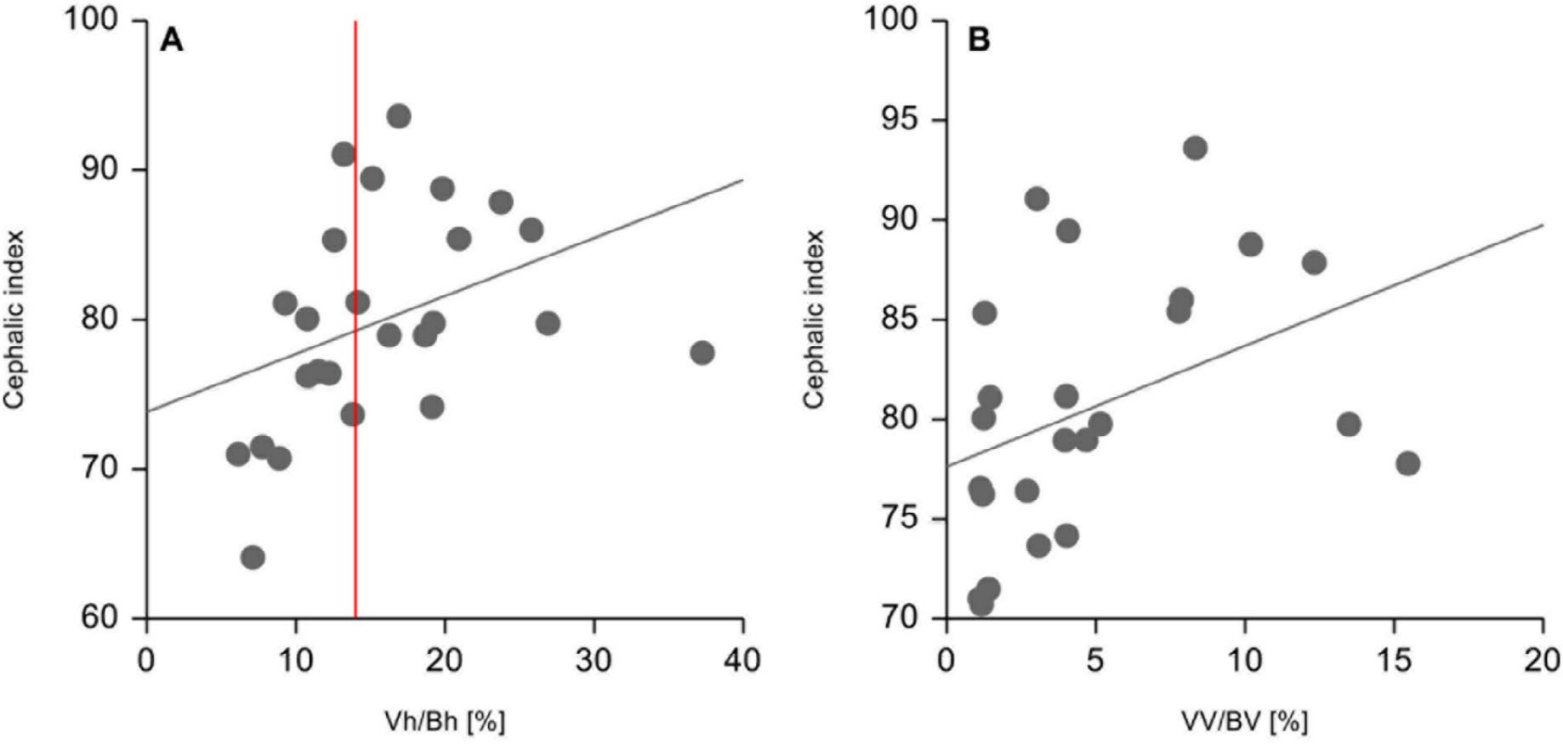
Scatter plots illustrating the correlation between cephalic index and (A) lateral ventricle to brain height ratio (Vh/Bh) (rho = 0.52, *p* = 0.002), (B) ventricle to brain volume ratio (VV/BV) (rho = 0.55, *p* = 0.001). Increased Vh/Bh ratios suggests increased lateral ventricles, whereas higher VV/BV ratios are consistent with a larger ventricular system. The degree of brachycephaly in a dog is directly reflected by its CI, with more brachycephalic dogs exhibiting higher cephalic index. The red line is set at the differentiation between normal‐sized lateral ventricles (0%–14%) and enlarged lateral ventricles (> 14%).

Correlation between ventricular size, assessed using Vh/Bh and VV/BV ratios, and the degree of brachycephaly, expressed by the cephalic index, was examined. Dogs with a higher cephalic index are considered more brachycephalic. Moderately higher Vh/Bh (rho = 0.52, *p* = 0.002) and VV/BV (rho = 0.55, *p* = 0.001) with a higher cephalic index were found (Figure 4) indicating that more severe brachycephaly is associated with larger lateral ventricles and larger internal CSF spaces. Furthermore, a lower body weight moderately correlated with a higher cephalic index (rho = −0.58, *p* = 0.002).

Correlations between cephalic index and CSF velocity parameters were assessed at the level of the mesencephalic aqueduct, FM, and C2 (Table 5). Lower peak diastolic velocity (rho = −0.61 and—0.58, *p* = 0.004 and 0.04, respectively), peak velocity (rho = −0.60 and—0.60, *p* = 0.005 and 0.03, respectively), difference between systolic and diastolic peak velocity (rho = −0.52 and—0.57, *p* = 0.02 and 0.04, respectively) and average velocity (rho = −0.63 and—0.62, *p* = 0.003 and 0.03, respectively) moderately correlated with higher cephalic index at the ventral and dorsal subarachnoid space of the FM. Moderately higher peak systolic velocity (rho = 0.45, *p* = 0.13) and lower maximum average velocity (rho = −0.60, *p* = 0.04 and 0.03) with higher cephalic index were found at the level of the dorsal subarachnoid space of the FM. Weakly higher peak systolic velocities (rho = 0.31, *p* = 0.19) and weakly lower maximum average velocities (rho = −0.37, *p* = 0.11) with higher cephalic index were found within the ventral subarachnoid space of the FM. In summary, increased brachycephaly is associated with decreased CSF velocity parameters at the level of the FM.

At the ventral subarachnoid space within C2, moderately lower peak diastolic velocity (rho = −0.40, *p* = 0.08), peak velocity (rho = −0.52, *p* = 0.02), difference between diastolic and systolic velocity (rho = −0.44, *p* = 0.05) and maximum average velocity (rho = −0.45, *p* = 0.05) with a higher cephalic index were found. A weak positive correlation between peak systolic velocity (rho = 0.25, *p* = 0.30) and cephalic index, and a weak negative correlation between average velocity (rho = −0.16, *p* = 0.50) and cephalic index were found within the ventral subarachnoid space at the level of C2.

At the mesencephalic aqueduct and dorsal subarachnoid space within C2, weak or no correlations were found between the CSF velocity parameters and cephalic index (all rho < 0.4 and *p* > 0.05).

### 3.5 Descriptive Analysis of the PC Sequences

In both groups, successful sagittal phase‐contrast measurements were achieved in approximately 50% of cases (Table 3). As a result, descriptive analysis was performed on images from nine dogs with enlargement of at least one internal CSF space and four without. An obstruction in CSF flow was interpreted by a lack of phase shift at any time point during systole and diastole.

In dogs with enlargement of at least one internal CSF space, a lack of phase shift ventral to the obex was observed in six of nine dogs. Among these, in five dogs, signal loss extended up to the FM, and in one dog, it extended up to the mid‐body of the C2 vertebra. In two of nine dogs, a loss of signal was detected at the level of the FM. Notably, one dog showed no loss of signal.

One dog without enlargement of the ventricular system showed no loss of signal, and one had signal loss extending from ventral to the obex to the FM. Another showed signal loss rostral to the FM. In the fourth dog, inadequate signal quality prevented evaluation.

## 4 Discussion

We detected differences in CSF velocity between small breed dogs exhibiting enlargement of at least one CSF space and those without enlargement at the level of the dorsal subarachnoid space within the FM. Our findings suggest that small breed dogs displaying enlargement of the ventricular system experience CSF flow disturbance at the level of the FM because differences in CSF velocity were not apparent at the mesencephalic aqueduct and C2. Increased CSF velocities at the mesencephalic aqueduct in brachycephalic dogs with both ventriculomegaly and internal hydrocephalus compared with normal controls were previously described [21]. Nevertheless, we found moderately higher CSF peak and maximum average velocities with enlargement of the ventricular system at the mesencephalic aqueduct. Whereas at the level of the FM and C2, an increase in ventricular size was associated with a decrease in CSF velocity.

Use of PC‐MRI identified CSF flow disturbances in humans diagnosed with obstructive hydrocephalus [12, 28, 29]. Patients with aqueductal stenosis had CSF flow with attenuated dynamics in the ventricular system [28, 29]. At the level of the stenosis, lower CSF mean and peak velocities are measured in patients with complete obstructive hydrocephalus [17, 29]. In patients with incomplete obstruction, turbulent CSF flow results in markedly accelerated CSF peak velocity at the level of the stenosis [17]. The notably lower CSF velocity parameters observed at the level of the FM in small breed dogs with enlargement of at least one CSF space suggest a disturbance in CSF flow caused by a complete, near‐complete, or functional obstruction, in which CSF flow is present but severely compromised. This obstruction of the CSF space subsequently could lead to enlargement of the ventricular system.

Recent hypotheses propose that CSF is produced and absorbed everywhere in the CSF system, challenging the traditional view that CSF accumulation occurs solely in front of an obstruction in the CSF pathway [30]. Instead, pathological processes such as inflammation, changes in osmotic pressure, or CSF dynamics are discussed as possible causes for the development of hydrocephalus [30, 31]. Nevertheless, a disruption in CSF shift from the cranial to spinal space because of obstruction around the spinal cord can cause an enlargement of the ventricular system [30]. In dogs, a proposed mechanism of the enlargement of the ventricular system is an obstruction of the CSF pathway at the level of the FM, a condition described as brachycephalic obstructive CSF channel syndrome (BOCCS) [32]. The lower CSF velocity parameters with Vh/Bh and VV/BV ratios we observed at the level of the FM support the hypothesis of BOCCS.

Increased ventricular size, indicated by higher Vh/Bh and VV/BV ratios, was noted in association with more severe brachycephaly, indicated by higher CI. A relationship of CI and ventricular size already has been observed in dogs [33] and Persian cats [34]. Constricted CSF pathways at the craniocervical junction are considered possible reasons for the high prevalence of ventricular system enlargement in brachycephalic dogs [4]. Small breed dogs are known to have anomalies of the craniovertebral junction [35, 36, 37]. Conditions such as Chiari‐like malformation and keyhole‐shaped FM potentially could result in disturbance of CSF flow at the level of the FM, as we determined using PC‐MRI. However, a comprehensive analysis of the craniocervical junction and associated anomalies was not carried out in our study.

A disruption of CSF flow because of obstruction at the level of the FM additionally is supported by the descriptive evaluation of the sagittal PC‐MRI images. Among small breed dogs with enlargement of at least one internal CSF space, six of nine dogs exhibited signal loss at the level of the FM. Such loss of signal typically indicates obstruction in CSF flow [22]. However, in 50% of dogs without enlargement of the ventricular system, a loss of signal at the level of the FM also was seen. Given the limited data, caution is advised when interpreting the sagittal PC‐MRI images of such dogs. Additional data are necessary for comparison.

## 5 Limitations

One major limitation of our study is the low number of dogs, especially for dogs without ventricular enlargement, because most small breeds show some degree of ventricular dilatation. Despite this, evidence of lower CSF velocity at the level of the FM was detected. Smaller differences at the mesencephalic aqueduct and C2 might have reached significance in a larger cohort.

Another major limitation is the fact that dogs with a clinical history and neurological conditions were used for our study. We did not recruit healthy small breed dogs for an MRI examination, but used dogs that underwent MRI of the brain or spine to evaluate various neurological and non‐neurological conditions. Even if dogs with mass effect with displacement of adjacent brain structures and those with brain atrophy were excluded, a disease‐related influence on CSF flow dynamics cannot be ruled out.

Differences in sex, age, and body weight should be taken into consideration when interpreting CSF flow [20]. Because the two groups did not differ in terms of mean age and sex distribution, only the significantly higher mean body weight of the dogs without enlargement of the ventricular system should be considered as an influencing factor. It is known in dogs that CSF peak velocity within the mesencephalic aqueduct and C2 is significantly higher with higher body weight [20]. Dogs with body weight < 10 kg have lower CSF peak velocities at the level of FM and C2 compared with dogs > 20 kg [20]. The difference in body weight between the two groups should be considered when interpreting our results, especially because lower body weight moderately correlated with a higher degree of brachycephaly in our study group.

Potential influence of skull shape on CSF velocity remains unexplored. The lower CI observed in dogs without enlargement of the ventricular system, indicating less brachycephalic skulls compared to dogs with enlargement, may have an impact on CSF velocity. Furthermore, the association between alterations in CSF flow, BOCCS, and ventricular enlargement is not yet fully understood. Changes at the craniocervical junction and skull shape can contribute to a decrease in CSF flow velocity, potentially leading to ventricular enlargement. Future studies are needed to investigate the relationship between CSF flow and skull shape, including abnormalities at the craniocervical junction.

Lastly, the low number of successful sagittal PC‐MR images (50%) is unsatisfactory. In theory, inadequate VENC settings or inaccurate placement of the measurement plane not aligned parallel to CSF flow could be potential reasons [13], although unlikely given thorough verification of all parameters. Likely, the low number of successful sagittal measurements is the result of difficulties with the peripheral pulse gating, because the peripheral pulse unit is not designed for attachment on the tongue of dogs. Another reason could be the neck position of the dogs [22]. Cerebrospinal fluid flow visualization and measurement at the dorsal subarachnoid space within the FM were more easily performed in dogs with more pronounced flexion of the head [22], but doing so was limited by the coils used in our study surrounding the head and neck.

## 6 Conclusion

In conclusion, ventricular enlargement in small breed dogs may be associated with or result in altered CSF flow dynamics, particularly decreased velocity at the craniocervical junction. This relationship, in turn, may reflect underlying structural changes, such as skull shape or craniocervical abnormalities. We therefore propose that enlarged intracranial ventricles in small breed dogs should be considered abnormal findings. Visualization of CSF flow and measurement of its velocity using PC‐MRI can be used as a clinical tool in dogs, helping to identify locations of CSF flow alterations and better understand CSF flow patterns in pathological conditions such as hydrocephalus, Chiari malformation, and syringomyelia.

Future studies are needed to gain a better understanding of changes in CSF dynamics in small breed dogs with enlargement of the ventricular system. We suggest recruiting a higher number of small breed dogs without clinical history, preferably dogs of one breed. Additionally, a thorough analysis of the craniocervical junction is recommended to assess the impact of craniocervical abnormalities on CSF flow dynamics.

## Disclosure

Authors declare no off‐label use of antimicrobials.

## Ethics Statement

This study was approved by the Cantonal Veterinary Office of Bern TVB Nr.: BE 47/2022 according to the Swiss national animal protection law. Authors declare human ethics approval was not needed.

## Conflicts of Interest

The authors declare no conflicts of interest.

## Supporting information


**Data S1:** Supporting Information.
